# Bilateral Upper Extremity DVT in a 43-Year-Old Man: Is It Thoracic Outlet Syndrome?!

**DOI:** 10.1155/2014/758010

**Published:** 2014-07-22

**Authors:** Hadoun Jabri, Sarbajit Mukherjee, Devang Sanghavi, Shyam Chalise

**Affiliations:** Department of Internal Medicine, Saint Joseph Hospital, Chicago, IL 60657, USA

## Abstract

Recurrent deep venous thrombosis, involving bilateral upper extremities, is an extremely rare phenomenon. We are presenting a 43-year-old man who was diagnosed with left upper extremity deep vein thrombosis (UEDVT) and was treated with anticoagulation and surgical decompression in 2004. 9 years later, he presented with right arm swelling and was diagnosed with right UEDVT using US venous Doppler. Venogram showed compression of the subclavian vein by the first rib, diagnosing thoracic outlet syndrome (TOS). He was treated with anticoagulation and local venolysis and later by surgical decompression of the subclavian vein. Bilateral UEDVT, as mentioned above, is an extremely rare condition that is uncommonly caused by TOS. To our knowledge, we are reporting the first case of bilateral UEDVT due to TOS. Diagnosis usually starts with US venous Doppler to detect the thrombosis, followed by the gold standard venogram to locate the area of obstruction and lyse the thrombus if needed. The ultimate treatment for TOS remains surgical decompression of the vascular bundle at the thoracic outlet.

## 1. Case Presentation

We are reporting a case of a 43-year-old male with a past medical history of DVT of the left subclavian vein as a result of thoracic outlet syndrome, diagnosed in 2004. He was treated with warfarin for 6 months and later underwent left first rib resection in 2006. He remained asymptomatic until May 2013 when he started complaining of right upper extremity discomfort and reddish discoloration that started after minimal muscle strain of his right arm while walking his dog. The patient had no neurological deficit in the right arm and did not complain of any weakness. He visited an urgent care center and had a US Doppler of the right upper extremity that confirmed the diagnosis of right subclavian vein thrombosis. Enoxaparin was given and he was transferred to our hospital.

The patient's past medical history was unremarkable except for the above. He was not taking any medication regularly. He was not a smoker, never used any illicit drugs, and only occasionally drank alcohol. He was a brick layer and was left handed.

His family history was significant for DM in both parents. No family history of clotting disorders was present.

His vitals were stable: BP 118/80, pulse 73/minute, temperature 97.7 F, and an oxygen saturation of 96% on room air. No signs of distress were noted.

Physical exam revealed edema and mild erythema of the right forearm as compared to the left forearm. No right arm weakness was appreciated and sensations were intact. The rest of the physical exam was normal.

Laboratory tests showed HB 14.2 g/dl (12–15.3 g/dl), platelet 271 k/mm (150–450 k/mm), and WBC 6.1 k/mm (4–11 k/mm). Coagulation profile was done and showed INR 1.1 (0.9–1.1), APTT 35 seconds (23–33 seconds), homocysteine level of 8.7, anticardiolipin IgG 0.8% (<20%), anticardiolipin IgM 2.7% (<20%), anticardiolipin IgA 1.8% (<12%), protein C 92% (>69%), and protein S 77% (>59%); prothrombin mutation was positive (heterozygous genotype) for G20210A mutation in factor II gene. Factor V Leiden mutation was negative.

Patient had a chest XR that showed a small right first rib (Figure 6). US Doppler of both lower extremities was done and was negative for deep vein thrombosis.

**Figure 1:**
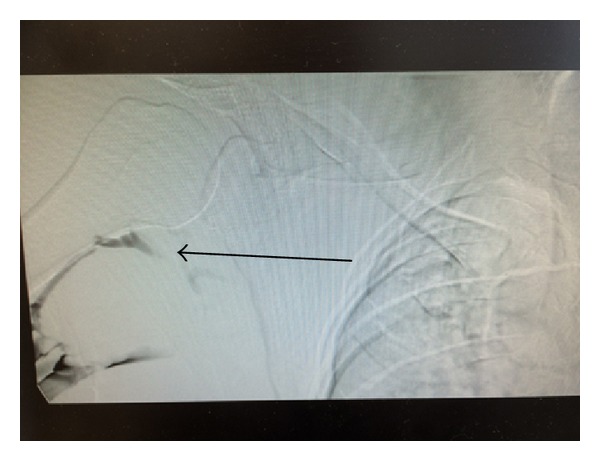
Another image showing extensive right upper extremity deep venous thrombosis on venogram.

**Figure 2:**
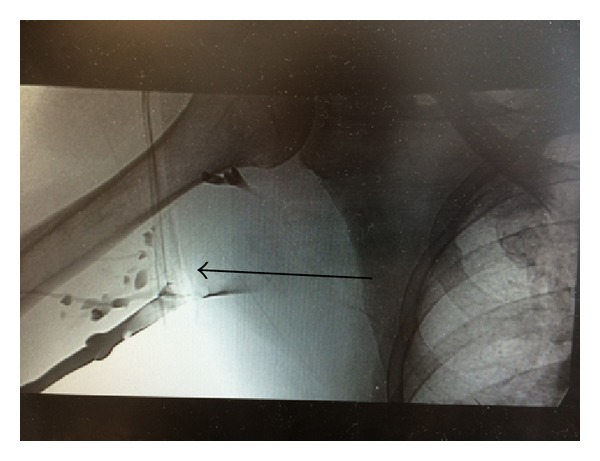
Extensive right upper extremity deep venous thrombosis on venogram.

**Figure 3:**
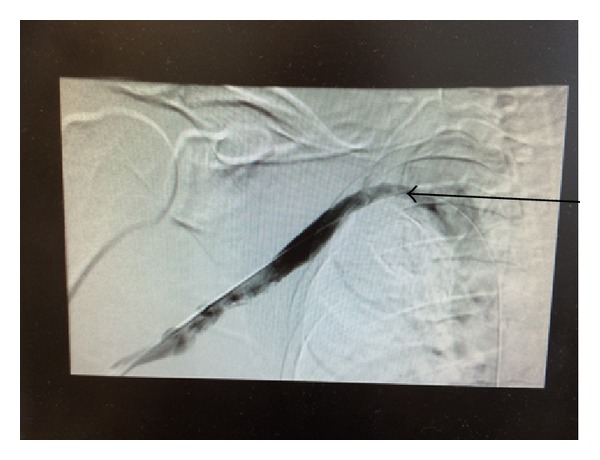
Chronic scarring of the right subclavian vein.

He was started on heparin infusion. After a day of anticoagulation, he had venogram that confirmed right subclavian vein thrombosis (Figures 1 and 2). Percutaneous transluminal balloon angioplasty of subclavian vein showed a narrowing at the level of the thoracic inlet that had an appearance of chronic scarring due to compression (Figure 3). Finally, a pharmacomechanical thrombolysis and thrombectomy were performed.

**Figure 4:**
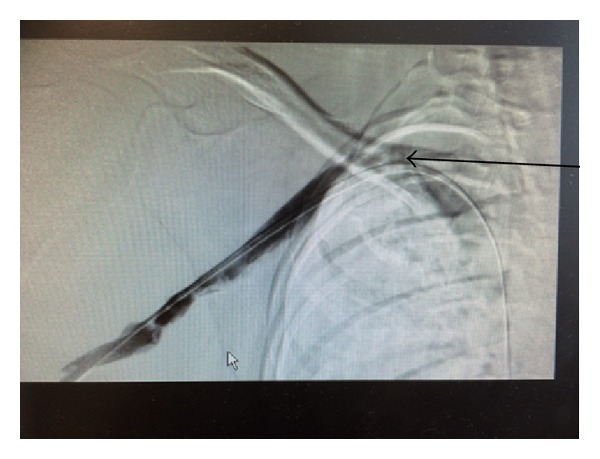
Successful venolysis. Chronic scarring of the right subclavian vein.

**Figure 5:**
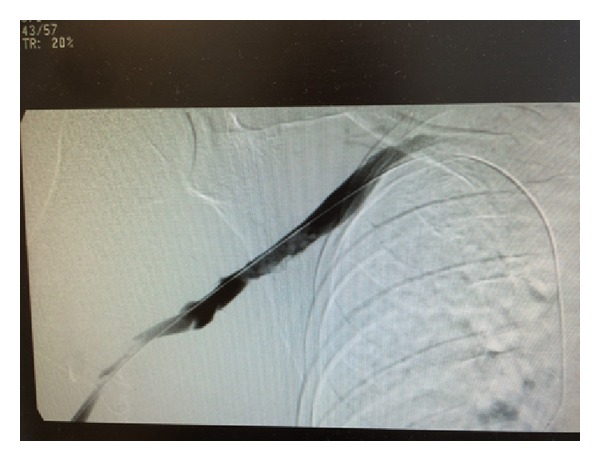
Successful venolysis.

**Figure 6:**
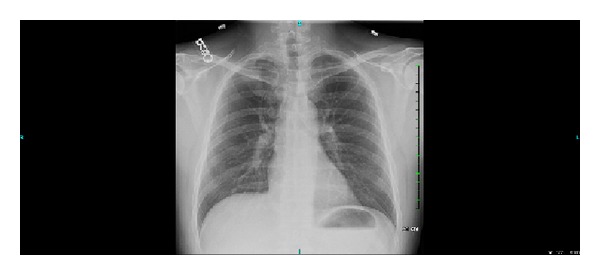
Chest X-ray of our patient before right first rib resection.

**Figure 7:**
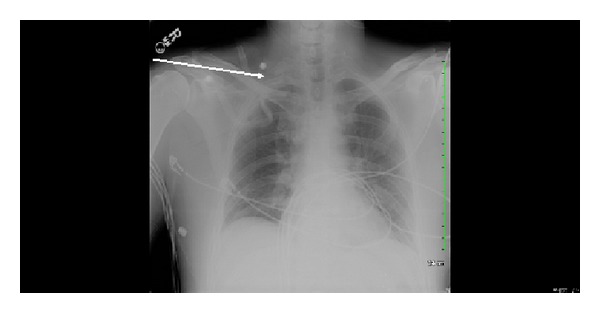
Chest X-ray of our patient after right first rib resection.

After the procedure, the patient was put on therapeutic dose of Enoxaparin and he was discharged home on that. A week later he returned to the hospital and had right first rib removal, complete anterior and middle scalenectomy, mobilization and protection of the brachial plexus with external neurolysis of C5-T1 roots, and external venolysis of the left subclavian vein (Figures 4 and 5). Patient was discharged from the hospital on Warfarin after he was seen by a hematologist. He remained asymptomatic and is following up as an outpatient. The procedure and anticoagulation were successful in treating his condition and no recurrence of his condition has been observed in a one-year follow-up (Figure 7).

## 2. Case Discussion

Deep vein thrombosis (DVT) of the upper extremity is diagnosed when a clot is visualized in the subclavian, axillary, or brachial vein.

Among all venous thrombosis patients, upper extremity deep venous thrombosis (UEDVT) compromises only 4–10% [1]. Even less common is the recurrent UEDVT. In a study that was done to evaluate the causes of recurrent UEDVT, the incidence was reported to be 4% as compared to 15% in lower extremity DVT [1]. All documented cases were in the ipsilateral upper limb [1].

Causes of UEDVT are various and are commonly related to immobility following surgery or casting, central venous catheterization, and strenuous muscular activity [2]. More remote but important cause includes thoracic outlet syndrome (TOS), in which nervous and vascular bundles are compressed at the base of the neck between the first or cervical rib and the clavicle [3]. According to our knowledge, all reported cases of DVT due to TOS are unilateral and this is the first case that showed bilateral DVT due to bilateral thoracic outlet syndrome.

Unless highly suspected, TOS can be easily missed as a correctable cause of subclavian vein compression and subsequently DVT. Chest X-ray can help raise the suspicion of TOS. In our patient, the recurrent DVT was associated with small right first rib on X-ray. This was a clue to do a venogram that showed compression of the subclavian vein. Invasive venogram is the gold standard to diagnose TOS causing DVT [4]. Magnetic resonant imaging (MRI) is a less invasive modality that helps identifying the anatomical relation between the veins and surrounding structures and may help in planning for surgery but is less sensitive than venogram in visualizing the thrombosis. Furthermore, venogram may help lysing the clot in the same session, as done in our patient.

Thrombophilia can predispose to DVT. Many mutations have been identified, out of which factor V Leiden and prothrombin gene mutations are the most common. Our patient was heterozygous for prothrombin G20210A mutation which has been associated with a 2.8-fold increased risk of venous thrombosis [5]. On the other hand, other studies have found that the association of this mutation with recurrent DVT remains unclear and controversial [6].

Treatment of UEDVT caused by TOS starts with anticoagulation as in DVT anywhere else. Thrombolytic therapy is usually tried by catheter infusion of alteplase in the vein itself [7, 8], while decompression of the thoracic outlet by first or cervical rib resection, division of anomalous musculotendinous bands, and scalenectomy remain the ultimate treatment [7, 8]. Many surgeons prefer doing venoplasty (open or percutaneous) as a step before surgery [4]. Pulmonary embolism (PE) as a complication of upper extremity DVT is rare as compared to lower extremity DVT [9]. A study done by Monreal et al. showed that PE occurring in cases of UEDVT is most likely to be related to UEDVT secondary to catheterization and primary UEDVT is rarely associated with PE [9].

## 3. Conclusion

1. Bilateral UEDVT is an extremely rare condition and should raise the suspicion for TOS.
2. TOS is usually caused by compression of the deep veins at the base of the neck.
3. Diagnosis of TOS causing UEDVT starts with US venous Doppler and is usually followed by venogram, which is considered the gold standard test.
4. Treatment starts with anticoagulation as in any DVT. Venolysis with local injection of a thrombolytic agent is preferred by many surgeons.
5. The ultimate treatment of TOS is the surgical release of the deep veins at the thoracic outlet.
